# Tracking Temporal Hazard in the Human Electroencephalogram Using a Forward Encoding Model

**DOI:** 10.1523/ENEURO.0017-18.2018

**Published:** 2018-05-08

**Authors:** Sophie K. Herbst, Lorenz Fiedler, Jonas Obleser

**Affiliations:** Department of Psychology, University of Lübeck, Ratzeburger Allee 160, 23552 Lübeck, Germany

**Keywords:** EEG, encoding models, implicit timing, SMA, temporal prediction

## Abstract

Human observers automatically extract temporal contingencies from the environment and predict the onset of future events. Temporal predictions are modeled by the hazard function, which describes the instantaneous probability for an event to occur given it has not occurred yet. Here, we tackle the question of whether and how the human brain tracks continuous temporal hazard on a moment-to-moment basis, and how flexibly it adjusts to strictly implicit variations in the hazard function. We applied an encoding-model approach to human electroencephalographic data recorded during a pitch-discrimination task, in which we implicitly manipulated temporal predictability of the target tones by varying the interval between cue and target tone (i.e. the foreperiod). Critically, temporal predictability either was driven solely by the passage of time (resulting in a monotonic hazard function) or was modulated to increase at intermediate foreperiods (resulting in a modulated hazard function with a peak at the intermediate foreperiod). Forward-encoding models trained to predict the recorded EEG signal from different temporal hazard functions were able to distinguish between experimental conditions, showing that implicit variations of temporal hazard bear tractable signatures in the human electroencephalogram. Notably, this tracking signal was reconstructed best from the supplementary motor area, underlining this area’s link to cognitive processing of time. Our results underline the relevance of temporal hazard to cognitive processing and show that the predictive accuracy of the encoding-model approach can be utilized to track abstract time-resolved stimuli.

## Significance Statement

Extracting temporal predictions from sensory input allows one to process future input more efficiently and prepare responses in time. In mathematical terms, temporal predictions can be described by the hazard function, modeling the probability of an event occurring over time. Here, we show that the human EEG tracks temporal hazard in an implicit foreperiod paradigm. Forward-encoding models trained to predict the recorded EEG signal from different temporal-hazard functions were able to distinguish between experimental conditions that differed in their buildup of hazard over time. These neural signatures of tracking temporal hazard converge with the extant literature on temporal processing and provide new evidence that the supplementary motor area tracks hazard under strictly implicit timing conditions.

## Introduction

Time provides the structure of our experience and is the basis of many cognitive processes, such as motor acts and speech processing. Even when we do not consciously track the passage of time, the extraction of temporal contingencies from the environment allows us to generate temporal predictions about the occurrence of future events (Nobre et al., 2007; Coull, 2009).

In mathematical terms, temporal predictions can be expressed by the hazard function, which models for any time point in a predefined interval the conditional probability of an event to occur, given it has not yet occurred. Crucially, temporal hazard depends on both elapsed time in the current situation and the observer’s expectation about possible durations, that is, the underlying distribution of durations. For example, when waiting at a traffic light, one might assume that waiting times vary in the range of several seconds and that any duration in that range is equally likely (i.e., assuming a uniform distribution of durations). In this case, the hazard for the light to turn green rises monotonically with elapsed time. Or, one could assume that some durations are more probable than others, for example because most traffic lights change from red to green at around 30 s (i.e., assume a distribution with one or more peaks resulting in a modulated hazard function). Little is known yet about how flexibly we extract statistical distributions of durations and use the resulting hazard functions to create temporal predictions, and which cognitive and neural processes are involved in this process.

Previous studies have shown that temporal hazard shapes behavioral responses (Karlin, 1966; Niemi and Näätänen, 1981; Tsunoda and Kakei, 2011; Tomassini et al., 2016) and is reflected in neural processing in monkeys (Akkal et al., 2004; Janssen and Shadlen, 2005; Jazayeri and Shadlen, 2015) and humans (Trillenberg et al., 2000; Praamstra et al., 2006; Cui et al., 2009; Bueti et al., 2010; Cravo et al., 2011; Jazayeri and Shadlen, 2015). For instance, Janssen und Shadlen(2005) tested whether monkeys learn to distinguish a unimodal from a bimodal duration distribution and showed that reaction times and recordings of single-neuron activity in the lateral intraparietal area correlated with the respective hazard functions of unimodal or bimodal duration distributions. In humans, Bueti et al. (2010), using functional MRI (fMRI), and Trillenberg et al. (2000), using electroencephalogram (EEG), showed that neural activity before target onset correlated with the respective hazard function. These studies used a so-called set-go-task, in which the participant has to withhold a response from a set-cue until the presentation of a go-cue, which enforces the use of temporal hazard. Trillenberg et al.’s participants were even informed about the underlying foreperiod probability distributions, which might have promoted the use of explicit timing strategies.

**Figure 1.**
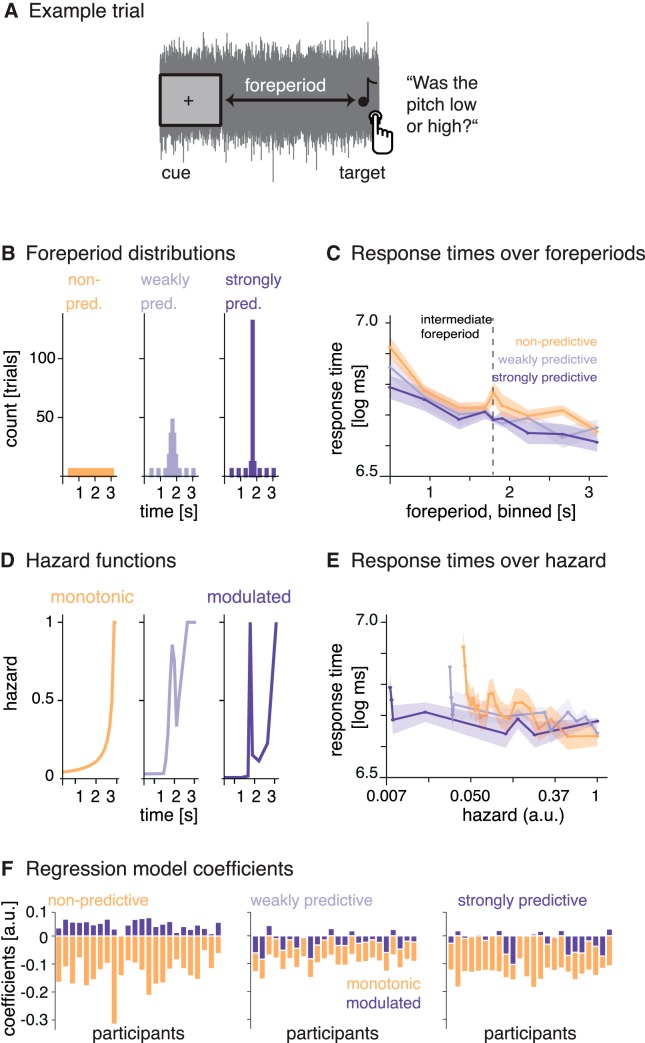
Paradigm and response times. ***A***, Example trial. On each trial, the simultaneous onset of the fixation cross and the noise served as a temporal cue. After a variable foreperiod interval, a single target tone was presented and participants had to judge its pitch as low or high. ***B***, Foreperiod probability distributions. Foreperiods for each block were drawn from one of three distributions: a uniform distribution (nonpredictive) and two normal distributions with larger and smaller standard deviations (weakly and strongly predictive). ***C***, Response times over foreperiods (binned): response times were longer at short foreperiods and shorter at long foreperiods. ***D***, Hazard functions resulting from the foreperiod probability distributions. ***E***, Response times over temporal hazard (i.e., the value of the hazard function at the time of target onset, on a log-spaced axis): response times were longest at the lowest hazard. Shaded surfaces reflect SEM. ***F***, Relative regression coefficients per participant obtained by modeling response times per condition (panels from left to right) with the monotonic and modulated hazard functions as regressors.

Here, our aim was to test whether observers use temporal hazard in a sensory task with no explicit incentives for timing. In contrast to explicit timing situations, in which participants are asked to provide an overt estimate of elapsed time, implicit timing does not require an overt judgment of time but assumes that temporal contingencies guide response behavior in a covered way (Coull and Nobre, 2008; Nobre and van Ede, 2018). Building on the earlier work, we were interested in whether human observers can distinguish between different levels of implicit temporal predictability. Instead of using duration distributions that differ with respect to the most likely time point at which an event is predicted to happen, we used three different unimodal probability distributions, all with equal mean. To vary the level of temporal predictability, we used either a uniform distribution (nonpredictive; mean duration = 1.8 s) or two Gaussian-shaped distributions with the same mean and larger (weakly predictive) and smaller (strongly predictive) standard deviations (see Fig. 1). Previously, we showed that the nonpredictive and strongly predictive conditions evoked distinguishable correlates of temporally predictive processing (Herbst and Obleser, 2017). Here, we asked whether the concept of temporal hazard, derived from the foreperiod distributions, can explain human behavior in an implicit foreperiod paradigm and whether it bears tractable signatures in the human EEG.

For the uniform foreperiod distribution, the hazard function rises monotonically toward the end of the range of possible intervals, but for the two Gaussian distributions, it is modulated and contains an earlier peak, too. To track the neural processing of temporal hazard over time, we measured response times and recorded neural activity with the EEG. We applied a forward-encoding model approach (following Lalor et al., 2006, 2009; see also O’Sullivan et al., 2015; Fiedler et al., 2017), using the hazard functions as time-resolved regressors to model the time-domain EEG data. To our knowledge, this is the first time that the encoding model approach has been applied to track the processing of an entirely abstract stimulus like temporal hazard, which is not related to the acoustic signal. Hence, a secondary aim of the presented approach was to proof the applicability of encoding models to track the processing of abstract stimuli in human time-domain EEG. Assessing the fit between modeled and measured time-domain EEG signals allowed us to quantify the representation of temporal hazard in the EEG, showing that the human brain distinguishes between different conditions of implicit temporal hazard.

## Materials and Methods

### Participants

A total of 24 healthy participants were tested (mean age 23.9 ± SD 2.2 years; 11 female, all right-handed), all reporting normal hearing and no history of neurologic disorders. Participants gave informed consent and received payment for the experimental time (**€**7 per hour). The study procedure was approved by the local ethics committee (University of Leipzig, Germany). Note that this paper reports extensive modeling and reanalysis of data that had been acquired for a study published earlier (Experiment II in Herbst and Obleser, 2017).

### Code and data accessibility

Publicly available Matlab toolboxes were used for the analyses (Fieldtrip, mTRF Toolbox: Crosse et al., 2016). The data and custom-written analyses scripts are available at the Open Science Framework: https://osf.io/qbhma/.

### Stimuli and paradigm

The EEG experiment was conducted in an electrically shielded sound-attenuated EEG booth. Stimulus presentation and collection of behavioral responses was achieved using the Psychophysics Toolbox (Brainard, 1997; Pelli, 1997). Responses were entered on a custom-built response box, using the fingers of the right hand for pitch judgment and the fingers of the left hand for a subsequent confidence rating. Auditory stimuli were delivered via headphones (Sennheiser HD 25-SP II) at 50 dB above the individual’s sensation level. Stimuli were pure tones of varying frequencies (duration 50 ms with a 10-ms on- and offset ramp), embedded in low-pass (5-kHz) filtered white noise. Sensation level was individually predetermined for the noise using the method of limits, and tone-to-noise ratio was fixed at –16 dB. Target tones varied in individually predetermined steps around a 750-Hz standard, which was itself never presented. Participants had to perform a pitch discrimination task on a single tone presented in noise: “Was the tone rather high or low?” (for an exemplary trial, see Fig. 1*A*), followed by a confidence rating on a three-level scale. The beginning of each trial was indicated by the simultaneous onset of the fixation cross and the noise. In 10% of all trials, participants received an additional explicit timing question (“Was the interval between the cue and the tone rather short or long?”) after the confidence rating. On average, 65% of these trials were answered correctly, showing that participants had processed the foreperiod duration and were able to retrieve it.

### Foreperiod distributions and hazard functions

Foreperiods ranged from 0.5 to 3.1 s (mean 1.8 s) and were drawn from three different probability distributions (shown in Fig. 1*B*). For the nonpredictive condition, 25 discrete foreperiods were drawn from a uniform distribution. We call this condition nonpredictive, because throughout the trial all tone onset times were equally likely. Nevertheless, because of the unidirectional nature of time, participants’ expectation for a target to occur should rise with elapsed time during these trials (as indicated by the hazard function, see below). For the two temporally predictive conditions, we drew foreperiods from two normal distributions with the same mean as the uniform distribution but varying standard deviations, to create a weakly predictive condition (SD = 0.15; resulting in five discrete foreperiods) and a strongly predictive condition (SD = 0.05; resulting in three discrete foreperiods). Furthermore, we added additional trials at three short (0.50, 0.93, 1.37 s) and three long (2.23, 2.67, 3.10 s) foreperiods to both predictive conditions (see foreperiod distributions in Fig. 1*B*), resulting in 25, 32, and 34 trials per block for the nonpredictive, weakly predictive, and strongly predictive conditions, respectively. The additional trials were included in all analyses, and foreperiods were presented in a counterbalanced manner.

Interstimulus intervals (ISIs) were drawn from a truncated exponential distribution (mean 1.5 s, truncated at 5 s). This way, we obtained maximally unpredictable ISIs (Näätänen, 1971), to prevent entrainment to the stimulation over trials. To obtain the hazard functions (depicted in Fig. 1*D*), we transformed the discrete values of the foreperiod probability distributions (including the additional trials). The hazard function is defined as

(1) $$H\left(t\right)=\frac{f\left(t\right)}{1-C\left(t\right)}$$

with *t* being time points throughout a trial *t* = 1…*T*, *f* the foreperiod probability distribution, and *C* the cumulative function thereof. For normalization purposes, we first replaced infinite values that occurred for *H*(*t*) when *C*(*t*) = 1 by the maximum of the remaining values of *H*(*f*), and divided all values by that maximum to achieve hazard values ranging between 0 and 1. The uniform foreperiod distribution from the nonpredictive condition results in a monotonic hazard function rising throughout the trial, indicating that if no tone has occurred yet, participants’ expectations for it to occur rises over time (Fig. 1*D*, left). The weakly and strongly predictive conditions show the same rise toward the end, but additionally contain a peak at the intermediate foreperiod, due to the predictability manipulation (Fig. 1*D*, middle, right). Of the two predictable hazard functions, we used only the hazard function from the strongly predictive condition for the following analyses. To obtain a maximal separation between the hazard functions from the nonpredictive and strongly predictive conditions for the encoding models, we subtracted the monotonic from the modulated hazard function, thus removing the rise toward the end of the trial from the modulated hazard function and keeping only the peak, as the most distinctive feature. We refer to this hazard function as the modulated hazard function.

**Figure 2.**
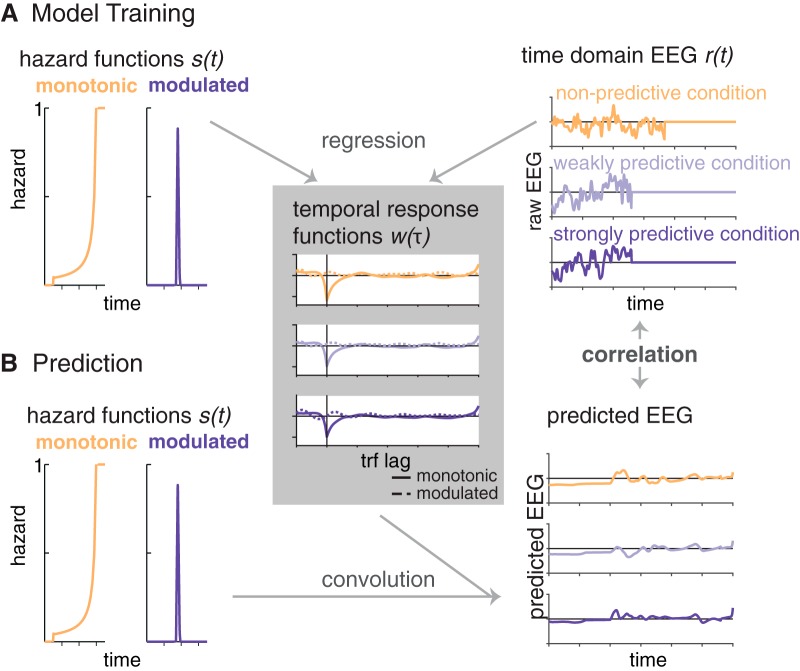
Schematic depiction of the encoding model approach. ***A***, One encoding model was computed per condition, by regressing the two hazard functions (*s* in Eq. 2) on the time-domain EEG data (*r* in Eq. 2; example trials are displayed, set to 0 at target onset. We obtained two temporal response functions (*w* in Eq. 2) per condition (right panel), one for the monotonic and one for the modulated hazard function. ***B***, In a second step, we predicted EEG signals (example trials displayed in the bottom right panel) from each of the three models by convolving the hazard functions with the temporal response functions. Correlating the predicted and original EEG signals allowed us to test which model provides the best fit with the original EEG data.

To be used as regressors in the encoding models fitted to the EEG data, the hazard functions had to match the sampling rate of the EEG data (200 Hz). To achieve this, we linearly interpolated the missing values. The resulting hazard functions are depicted Fig. 2*A*.

### Procedure

At the beginning of the session, participants were briefed about the pitch discrimination task and the EEG recording. There was no mention of any aspects of timing before testing. After EEG preparation and the measurement of the individual sensation level, participants performed a training of 38 trials (25 for the pitch discrimination only plus 13 with the confidence rating added). During training, foreperiods were drawn from a uniform distribution to not induce any temporal predictions. Then, six times three condition blocks were presented in random order: each condition was presented once during the first and once during the second half of the experiment (three consecutive blocks per condition with random order between condition blocks, separated by breaks of self-determined length (60 s at minimum). There were 546 trials in total. After the experiment, participants were debriefed and asked whether they had detected the foreperiod manipulation. No participant spontaneously reported the predictability manipulation we applied. In a second question, the experimenter asked participants whether they detected any differences in the distributions of short and long foreperiods between blocks. Two participants stated they did detect a difference, which could be a hint that they noticed the predictability manipulation. In total, the experimental session lasted ∼2.5 h, including EEG preparation.

### EEG recording and preprocessing

Electroencephalographic data were continuously acquired from 68 electrodes (Ag–AgCl), including 61 scalp electrodes (Waveguard, ANT Neuro), one nose electrode, and two mastoid electrodes. The electro-oculogram was acquired to record eye movements, with two electrodes placed horizontally to each eye and one placed vertically to the right eye. A ground electrode was placed at the sternum. All impedances were set <10 kΩ. The nose electrode served as reference during recording. The data were acquired with a sampling rate of 500 Hz and a hardware-implemented passband of DC to 135 Hz (TMS International). EEG data were analyzed using the Fieldtrip software package (versions 20160620, 20170501) for Matlab (MATLAB 2016a, 2017a). First, we applied high- and low-pass filtering to the continuous data using filters from the firfilt plugin (Widmann et al., 2015). The high-pass filter was a causal (one-pass-minphase) firws filter at 0.1 Hz with a transition bandwidth of 0.2 Hz. The low-pass filter was a causal firws (one-pass, zero-phase) filter, with a 100-Hz cutoff and 3-Hz transition bandwidth. After filtering, data were re-referenced to linked mastoids, down-sampled to 200 Hz, and demeaned. Next, the data were epoched around the cue (onset of fixation cross and noise) with a 4-s prestimulus and a 6.5-s poststimulus interval. Artifact correction was performed in three steps: visual inspection and removal of trials with excessive artifacts occurring at all channels (on average 22.7 of 546 trials) and marking of bad channels that were excluded from the independent component analysis (ICA) and interpolated afterward (one each for four participants, zero for all others); removal of eye blinks and muscular artifacts by visually inspecting and then removing ICA components (rejecting on average 26.6 of 64 components); and automatic removal of trials with activity exceeding ±150 µV (on average 25.8 trials). For the encoding models, the epoched data were low-pass filtered (sixth-order, two-pass butterworth filter, 25-Hz cutoff).

### Analyses of response times

All behavioral analyses were performed in R (version 3.3.0, R Core Team, 2016), using linear mixed-effect models from the lme4 package (Bates et al., 2015), as well as plotting functions from the ggplot2 package (Wickham, 2009). Response times were log-transformed, and the first block per condition was removed from the analyses to allow participants to adapt to the new condition. Trials with response times less or more than 2.5 individual standard deviations were removed as outliers. To generally assess whether hazard affects response times, we computed a linear mixed-effect model with one regressor of interest (fixed effect), namely the value of the hazard function for the respective condition at the time point of the occurrence of the probe (0-centered), plus we modeled a random intercept and random slope for the effect of hazard over participants.

Second, to test the differential fit for each of the two hazard functions in each condition, we computed one linear mixed-effect model per condition, using only the data from that condition and both the monotonic and modulated hazard functions additively as fixed effect regressors, plus a random intercept and random slopes for both hazard regressors over participants.

To obtain *F*- and *p*-values for the fixed effects, we used the summary function from the lmerTest package (Kuznetsova et al., 2016). As an estimate of effect sizes, we report conditional and marginal *R* ^2^ values (Nakagawa and Schielzeth, 2013; Johnson, 2014), which indicate the variance explained by the full model and by the fixed effects, respectively (obtained from the MuMIn package, Bartoń, 2017).

### EEG analyses: forward-encoding models

To test whether the time-domain EEG signal tracks temporal hazard, we used a forward-encoding model approach as previously described by Lalor et al. (2006; 2009), in which a time-resolved regressor (such as a speech envelope, or in our case the hazard function), is used to predict a time-resolved neural signal (EEG or MEG time domain or frequency data) for a range of negative and positive time lags between the two signals for each trial, using ridge (or L2-penalized) regression (Hoerl and Kennard, 1970; see also O’Sullivan et al., 2015; Biesmans et al., 2017; Fiedler et al., 2017).

The approach is conceptually similar to cross-correlation, but more robust to overfitting owing to usage of regularized regression. For these analyses, we used the Matlab-based multivariate temporal response function (mTRF) toolbox (version 1.3, Crosse et al., 2016). The resulting regression weights over lags, termed temporal response function (TRF), *w*(τ,*n*), can be understood as a filter with which the stimulus *s*(*t*) is convolved to obtain the response *r*(*t*,*n*) (Crosse et al., 2016):

(2) r(t,n)=∑w(τ,n)s(t−τ)+ε(t,n).

Here, 1…*T* reflects sampling points of *r* (EEG response, measured at *n* channels), and *s* (stimulus) over time, and τ the samplewise time lag between *s* and *r*. For instance, the EEG signal might show a relation to the sensory stimulus not at lag 0 (i.e., simultaneously), but shifted in time, for example with a lag of 100 ms relative to the stimulus signal. Similar to correlation coefficients, the relation can be positive or negative. ε(*t*,*n*) reflects the residual errors not explained by the model. *w*(τ,*n*) can be obtained by minimizing the mean squared error, which is implemented via regularized ordinary least squares:

(3) $$W={\left(S^{T}S+\lambda mI\right)}^{-1}S^{T}R,$$

where *S* is the stimulus over time points *t* (rows), shifted over lags τ (columns). A regularization parameter (the ridge parameter) is introduced to prevent overfitting. λ is multiplied with *m*, the mean of the diagonal elements of *S^T^S*, and with the identity matrix *I*, and added to the covariance matrix *S^T^S* (Biesmans et al., 2017). Here, we used λ = 4, which we had found to be the minimal value to obtain stable TRF by visually inspecting the ridge trace: the peak value of the TRF (at lag 0) over different values for λ.

To illustrate the overall shape of the temporal response functions in an unbiased manner, before selecting specific channels and time points, we computed global field power over channels.

### EEG encoding models of auditory events

**Figure 3.**
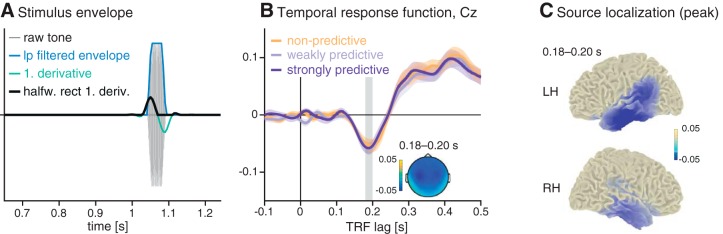
Encoding models trained on the target-onset envelopes. ***A***, Preparation of stimulus envelopes: stimulus vectors were computed by inserting the target tone into a vector of zeros at the time of its occurrence, extracting the envelope and subsequently computing the halfwave rectified first derivative (black line). ***B***, Temporal response functions to the target tone for all three experimental conditions. The inset shows the topography at the negative peak (0.18–0.20 s). ***C***, Source localization for the negative peak of the temporal response function (averaged over conditions, 0.18–2.0 s), suggesting auditory generators.

In a first step, to ensure that the forward modeling approach works even for our relatively short epochs, we computed TRF for the encoding of the auditory events, that is, the target tones embedded in ongoing noise. To test for the encoding of the target tone onset in the EEG signal, we created for each trial a time-domain stimulus vector (from 0.5 to 3.5 s after cue-onset) by inserting the target tone into a vector of zeros, at the time point when it occurred on that trial (as in Fiedler et al., 2017). We computed stimulus envelopes by taking the absolute values of the analytic signal, then downsampled the signal to the sampling frequency of the EEG data (200 Hz), low-pass filtered (sixth-order, two-pass Butterworth filter, 25-Hz cutoff), and took the halfwave rectified first derivative (see Fig. 3).

### EEG encoding models of temporal hazard

To assess whether and how the brain tracks temporal hazard, we computed temporal response functions by training three encoding models with the temporal hazard functions as regressors (see schematic depiction in Fig. 2). We trained one encoding model on the data of each of the three conditions (nonpredictive, weakly predictive, and strongly predictive) by using the monotonic and modulated hazard functions jointly as regressors. This way, we obtained per encoding model two TRFs: one TRF reflecting the regression weights for the monotonic hazard function, and one TRF reflecting the regression weights for the modulated hazard function. Second, to test whether the EEG data from each condition encodes the temporal hazard applying to each of the three conditions, we predicted the EEG responses from each of the three models and tested the fit of the predicted with the original EEG responses from all three conditions by computing Pearson correlation coefficients between predicted and original data.

Encoding models were computed for single trials, allowing us to take into account the variable intervals between cue and target. Only trials with foreperiods longer than 0.65 s were used and cut from 0.5 s after cue onset (to remove the cue-evoked activity). Importantly, we omitted the response to the target tone by setting the EEG signal to 0 after the time point of target onset on the given trial, because temporal hazard is relevant only until the occurrence of the expected event. Both the EEG data and hazard function vectors were low-pass filtered (using a sixth-order two-pass Butterworth filter with a cutoff frequency of 25 Hz). The hazard functions (also cut from 0.5 s after cue-onset) were normalized by dividing through their maximum, and the EEG data for each trial were *z*-scored over the temporal dimension. The lags for the temporal response function were chosen as –0.2 to 0.6 s.

To predict EEG data, we used a leave-one-out procedure over trials, in which we predicted EEG data for one trial by using the average TRF from all other trials in that condition. To test whether the models are able to distinguish between the data from the three conditions, we also predicted EEG data from the models trained on the other two conditions. We thus obtained for each condition three data vectors (per trial, participant, and channel) from the models trained on the nonpredictive, weakly predictive, and strongly predictive conditions, respectively. Next, we computed per-trial Pearson correlation coefficients between the predicted EEG data and the original data to test which of the three models provides the best fit with the original condition data (referred to as “testing condition” in the figures). Correlation coefficients were Fisher *z*-transformed. To assess whether the correlations were different from zero, that is, whether the models had any predictive power, we computed parametric 95% confidence intervals for the true correlation based on the *T*-distribution.

Then, to assess relative fits between the models trained on the data from the three different conditions, we computed a “robust” index of Fisher *z*-transformed correlations for each trial. To deal with different signs of individual correlation values, we applied the inverse logit transform to individual correlation values and then computed the index as the normalized difference between the correlation obtained from the model trained on the data from the nonpredictive condition and the correlation obtained from training on the data from the strongly predictive condition. A correlation index larger than zero indicates a relatively better fit obtained by the model trained on the nonpredictive condition, whereas a correlation index smaller than zero indicates a relatively better fit obtained by the model trained on the strongly predictive condition. A correlation index of zero would indicate that both models perform equally well. To test the significance of the differences in correlation indices between conditions, we performed a second-level cluster permutation test on the indices obtained for each participant over all electrodes (two-tailed, α = 0.025, 1000 permutations).

### Source localization

To localize the peaks of the temporal response functions from the sensor-level analyses, we performed an additional source localization analysis. For the coregistration of the EEG electrode positions we used, due to the nonavailability of individual anatomic models for our participants, a standard electrode layout, a standard MRI template, and a standard head model (based on the boundary-elements method) from FieldTrip software (Oostenveld et al., 2003). Reconstruction of sources from EEG data based on template anatomical models has been successfully performed by previous studies from our own group and others (for instance, Praamstra et al., 2006; Bendixen et al., 2014; Strauß et al., 2014; Helfrich et al., 2017).

Data were re-referenced to the average of all channels, and individuals’ lead field matrices were calculated with a 1-cm grid resolution. For each participant, a spatial filter was computed by applying linearly constrained minimum variance (LCMV; Van Veen et al., 1997) beamformer dipole analysis to trial-averaged data (dipole orientation was not fixed). Single trials where then projected into source space, and a principle components analysis was computed on single-trial 3D time course data (*x*,*y*,*z* direction) to extract the dominant orientation (first component). Then, temporal response functions were computed on the single-trial data from each source, exactly as described for the sensor level data. For visualization, the source-level peaks of the average temporal response functions over participants were interpolated and projected onto a standard MNI-brain. To illustrate the temporal response functions in supplementary motor area, we extracted single-voxel activity from the labels from the AAL atlas (left and right supplementary motor area; SMA), implemented in fieldtrip (Tzourio-Mazoyer et al., 2002) and averaged over all voxels in the label.

### Control analysis on resting-state data

As a control analysis, we computed the encoding models on time-domain EEG snippets from a resting-state EEG data set. We used 3 min of eyes-open resting-state recordings that had been acquired from 21 participants in an independent study (Wöstmann et al., 2015, 2014). We applied the same high- and low-pass filters to these data as described above, re-referenced to linked mastoids, downsampled to 200 Hz, and used ICA to remove eye movements. Then, for each of the participants, we randomly selected snippets from the resting-state data and replaced the original trial data with those snippets. To simulate 24 participants, we performed a second random selection for 3 of the 21 resting-state participants. Encoding models were computed for these data, exactly as described for the original task data.

## Results

### Temporal hazard affects response times

To test whether response times vary with temporal hazard, we first computed an overall linear mixed-effects model jointly for all conditions, with hazard as continuous regressor. As hazard, we input the value of the hazard function used for that condition at the time point of target occurrence (as depicted in Fig. 1*E*). Response times to a given target were strongly influenced by hazard at target occurrence, shown by a significant main effect of hazard (*F*(1,23.1)= –4.2, *p* < 0.001; conditional *R* ^2^ = 0.445, marginal *R* ^2^ = 0.003).

Furthermore, we tested whether response times indicate a distinction between the three different hazard functions used, relating to the approach used by Janssen and Shadlen (2005) and Bueti et al. (2010). To this end, we separated the data from the three conditions and used as regressors the hazard functions from the nonpredictive (monotonic hazard function) and strongly predictive conditions (modulated hazard function). Note that the weights obtained for each regressor indicate the influence of this particular regressor on the data when all other regressors are kept constant, that is, the unique variance it explains. We expected that if observers updated their temporal expectations between conditions, the relative weights obtained for the two hazard functions should differ between conditions.

For all three conditions, we obtained a negative weight for the monotonic hazard function, showing that response times decreased with rising hazard. The effect for the monotonous hazard function was significant for all conditions (marginally only in the weakly predictive condition; *p* values are given in Table 1). On average, the weights for the modulated hazard function were positive in the nonpredictive condition but negative in the two predictive conditions, but the modulated hazard function did not produce a significant effect in any condition.

**Table 1.** Linear mixed-effect model of response times.

Condition	Nonpredictive	Weakly predictive	Strongly predictive
Monotonic hazard function	–0.14 (0.06); *p* < 0.001	*–0.07 (0.04); p = 0.06*	–0.11 (0.04); *p* < 0.01
Modulated hazard function	0.04 (0.04); *p* = 0.29	–0.03 (0.02); *p* = 0.18	–0.03 (0.02); *p* = 0.27
Conditional *R* ^2^	0.448	0.442	0.429
Marginal *R* ^2^	0.007	0.001	0.002

Results of the three linear mixed-effect models computed separately to predict response times from the nonpredictive and weakly and strongly predictive conditions with the monotonic and modulated hazard functions. The first value in each cell gives the parameter estimate (i.e., the unit change in log response times by this factor when keeping all other factors constant) and its standard error in parentheses. *P*-values indicate which hazard functions significantly correlated with the response times from the respective condition. Conditional and marginal *R* ^2^ values indicate the variance explained by the full model, and by the fixed effects, respectively.

As shown in Fig. 1*F*, the relative contributions of the modulated and monotonic hazard functions changed over conditions, as indicated by an ANOVA computed on single subject’s ratios between the weights for the modulated and monotonic hazard functions (*F*(2,69) = 19.98, *p* < 0.0001). *Post hoc* tests revealed significant differences between the nonpredictive and both predictive conditions (*p* < 0.001, fdr-corrected).

In sum, these results show that while the monotonic hazard function affected response times in all conditions, the relative contribution of the monotonic and modulated hazard functions differed between conditions, suggesting that response times do distinguish between the different hazard functions.

### Temporal response functions of auditory stimulus encoding

To assess the neural encoding of target tones and ensure the overall applicability of forward-encoding models to these data, we trained encoding models on the time-domain EEG data from all conditions, using target-onset envelopes as regressors. The resulting trial-averaged TRFs (shown in Fig. 3*B*) show a negative deflection around lag 180 ms, whose topography and source reconstructions suggests sources in auditory areas (Figure 3C). The TRF showed no condition differences within the range of lags applied. This initial analysis proved that even with relatively short epochs, the approach can reveal the encoding of auditory information.

### Temporal response functions reveal the neural tracking of temporal hazard

**Figure 4.**
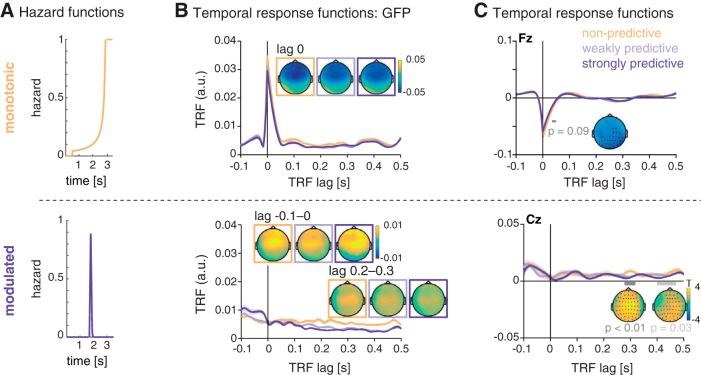
Temporal response functions (TRFs) for the encoding of temporal hazard, separately for the different hazard components. ***A***, Hazard functions, as used as regressors for the encoding models. Top: monotonic hazard function (orange); bottom: modulated hazard function (violet). ***B***, Global field power (GFP) of the TRF for the monotonic (top) and modulated (bottom) hazard function. Shaded areas indicate SEM over participants. Topographies show the scalp distribution of the TRF at lag 0 for the monotonic hazard function and lags –0.1 to 0 s and 0.2 to 0.3 s for the modulated hazard function. ***C***, Average temporal response functions for the monotonic (top, electrode Fz) and modulated (bottom, electrode Cz) hazard function. Insets show statistically significant differences between the TRF for the three conditions.

Our main analysis sought to test whether the time-domain EEG signal contains signatures of temporal hazard. We computed one encoding model per condition (per participant, trial, and channel), using as regressors the monotonic and modulated hazard functions obtained from the nonpredictive and strongly predictive conditions (Fig. 4*A*). For each encoding model (i.e., per experimental condition), we obtained two TRFs, one for each of the two hazard functions (as shown in Fig. 4*B*). The TRF obtained from the monotonic hazard function showed a peak at lag zero in global field power, which results from a negative deflection with a fronto-central scalp distribution in all conditions. A cluster-permutation test on the single-subject TRFs (from –0.05 to 0.05 s) revealed a marginally significant greater negativity for the peak for the TRF trained with the data from the nonpredictive compared to the predictive condition (0.035–0.05 s; *p* = 0.09).

The TRF for the modulated hazard function showed no peak at lag zero but, in global field power, a stronger positivity at negative lags and a rather a sustained difference between conditions starting at a lag of ∼150 ms. However, significant differences (testing for all latencies from –0.1 to 0.5 s) were found only for the later lags: between 0.28 and 0.32 s (*p* < 0.01) and 0.42 and 0.47 s (*p* = 0.03).

### Encoding models of temporal hazard distinguish between experimental conditions

**Figure 5.**
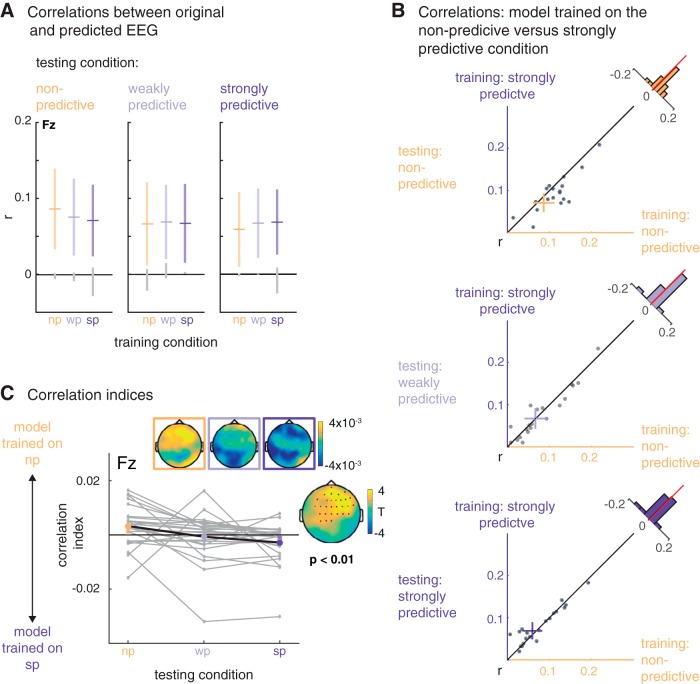
Relative model fits. ***A***, Mean (horizontal bars) and 95% confidence intervals (colored vertical bars) for the correlation between the original EEG data from nonpredictive (np), weakly predictive (wp), and strongly predictive (sp) conditions (testing condition, displayed in the horizontal panels) and the predicted EEG from the models trained on the nonpredictive and weakly and strongly predictive conditions (on the *x*-axis and color-coded). The gray vertical lines display the confidence intervals obtained from the resting-state data. ***B***, Scatterplots, displaying for each condition (testing condition, vertical panels) single participants’ correlations between the data from that condition with the data predicted by the model trained on the nonpredictive (*x*-axis) versus strongly predictive (*y*-axis) conditions. ***C***, Relative correlation indices: positive and negative values indicate better fits between the original data per condition (*x*-axis) and the data predicted by the model trained on the nonpredictive and strongly predictive condition, respectively. The insets show the topographic distribution of the indices and the *t* values for the statistical comparison of the nonpredictive and the strongly predictive condition.

To assess the relative fit of the three encoding models trained on the data from each condition, we predicted EEG data based on each of the three models (see example in Figure 2B). As a measure of prediction accuracy, we then correlated each trial of predicted EEG with the original EEG data. As shown in Fig. 5*A*, predicted and original EEG data correlated positively, around *r* = 0.07 on average. As shown by the confidence intervals (colored vertical bars in Fig. 5*A*), the correlations were significantly different from zero (and from the correlations obtained with the resting-state data, gray vertical bars in Fig. 5*A*).

To assess the relative fits of the three different models trained on the data of the three conditions, we computed correlation indices, contrasting for each trial the fit of the original EEG data with the predicted EEG signals from the models trained on the nonpredictive versus strongly predictive conditions. The resulting index is positive when the original data fits better with the predicted data from the model trained on the nonpredictive condition, and negative when the predicted data fits better with the data from the strongly predictive condition. As shown in Fig. 5*C* for the nonpredictive condition the average index showed relatively better fits for the model trained on the nonpredictive condition with the original data from that condition, and respectively between the test data from and the model trained on the strongly predictive condition. For the weakly predictive condition, the index is at approximately zero, indicating no conclusive evidence for either model. A two-level permutation cluster test on individual participants’ correlation indices per trial confirmed a significant difference between the correlation indices from the nonpredictive versus the strongly predictive condition (*p* = 0.002). In sum, the differences in correlation indices revealed that the three encoding models are sensitive to condition differences in the time-domain EEG data.

### Encoding of instantaneous temporal hazard in the SMA

**Figure 6.**
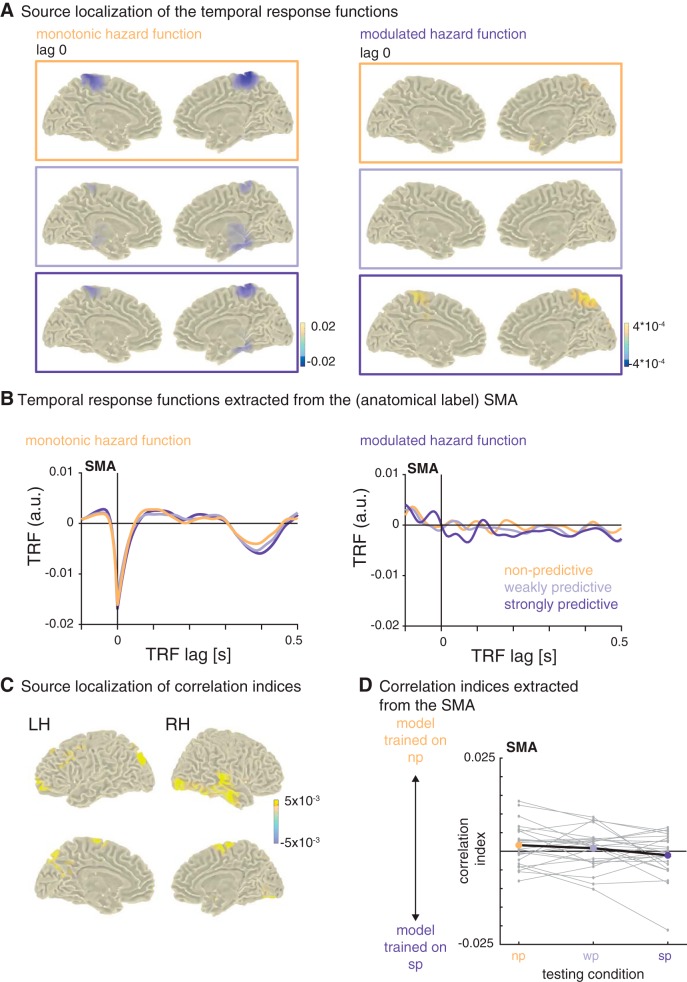
Source localization of the temporal response functions. ***A***, Peak sources of the temporal response functions at lag 0 (the transparent overlay on the color bar indicates the value at which the plots were masked). The strongest negative activity was localized to the bilateral SMA and adjacent regions. ***B***, Temporal response functions extracted from the bilateral SMA. ***C***, Sources of relative correlation indices: difference between testing conditions nonpredictive and strongly predictive (*x*-axis in ***D***). ***D***, Relative correlation indices, extracted from the bilateral SMA: positive and negative values indicate better fits between the original data per condition (*x*-axis) and the data predicted by the model trained on the nonpredictive and strongly predictive condition, respectively.

To assess the anatomic sources of the temporal response functions, we computed the encoding models on EEG data projected into source space (see Fig. 6). This analysis revealed that the negative peaks of the TRF for the monotonic hazard functions found in the sensor space data originate in the bilateral SMAs and the adjacent paracentral lobule (Fig. 6*A*, left).

There was no clear peak for the TRF for the modulated hazard functions in the sensor space data. In source space, the strongest activation at lag 0 was originating from the precuneus, but only in the strongly predictive condition (Fig. 6*A*, right). To *post hoc* visualize the TRF from the SMA over lags, we then extracted activity from the SMA in source-space for both hazard functions and all three conditions. The TRF for the modulated hazard function showed a stronger positivity from lags –0.05 to 0 s and an adjacent negativity (lags 0 to 0.1 s) only for the strongly predictive condition (Fig. 6*B*, *C*). Correlations and correlation indices computed on the data extracted from the SMA showed a pattern similar to that of the sensor-level data, namely better fits between the model trained on the nonpredictive condition with the data from that condition, and, respectively, better fits between the model trained on the strongly predictive condition with the data from that condition (Fig. 6*C*,*D*), but the difference was not significant (*T*(23) = 1.64, *p* = 0.11), indicating that the SMA is not the only region contributing to the distinction between the different hazard functions. The peak sources for the positive differences in correlation indices (shown in Fig. 6*C*, *D*) were in the bilateral SMA, precuneus, and medial temporal lobes.

### Control analysis: encoding models on resting-state data

**Figure 7.**
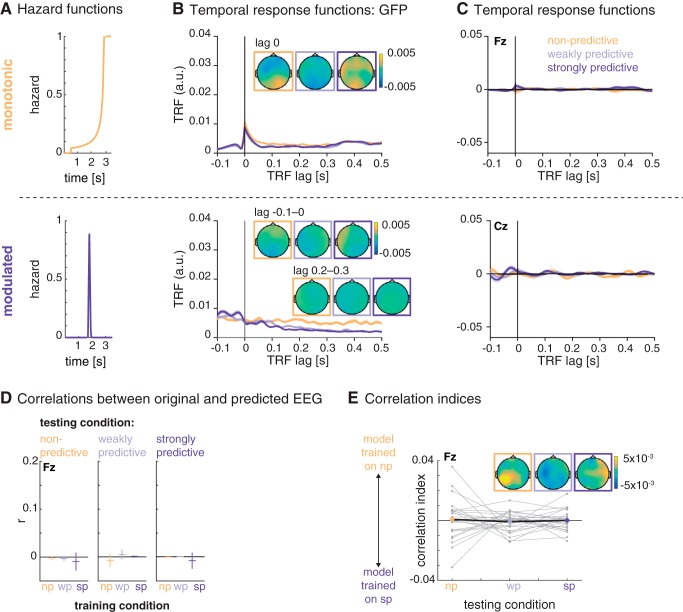
Control analysis on resting-state data. ***A***, Hazard functions, used as regressors for the encoding models. Top, monotonic hazard function (orange); bottom, modulated hazard function (violet). ***B***, Global field power for the monotonic (top) and modulated (bottom) hazard function. Shaded areas indicate SEM over participants. Topographies show the scalp distribution of the TRF at lag 0 for the monotonic hazard function and lags –0.1 to 0 and 0.2 to 0.3 s for the modulated hazard function. ***C***, Average temporal response functions for the monotonic (top, electrode Fz) and modulated (bottom, electrode Cz) temporal response functions. ***D***, Mean (horizontal bars) and confidence intervals (vertical bars) for the correlations between the original resting-state EEG data (testing condition, displayed in the horizontal panels) and the predicted EEG from the models trained on the nonpredictive and weakly and strongly predictive conditions (on the *x*-axis and color-coded). ***E***, Relative correlation indices: positive and negative values indicate better fits between the original data per condition (*x*-axis) and the data predicted by the model trained on the nonpredictive and strongly predictive condition, respectively.

To assess the validity and specificity of the encoding model approach, we computed the same encoding models (i.e., using the same set of hazard function regressors) on time-domain EEG snippets from a resting-state data set. Fig. 7 shows the TRFs obtained from these data. The TRF for the monotonic hazard function has a peak in global field power at lag 0 s, albeit weaker than with the original data and with much less clear topographies. The TRF for the modulated hazard function showed a sustained difference in global field power at later lags.

The (at least partial) resemblance between the TRF obtained for the original condition data and the resting-state data confirms that a forward-encoding model can pick up unspecific activity, and that the regressors used for the model (here, the hazard functions) do influence the shape of the TRF to a substantial degree. Most importantly, however, when predicting EEG data from the three models trained on the resting-state data and correlating the predicted and original resting-state data, the resulting correlations did not differ significantly from zero, and the correlation index did not differ between conditions (see Fig. 7*D*, *E*). This control analysis rules out the possibility that the results obtained with the actual task EEG data were due to generic properties of EEG and encoding models only. In particular, one could worry that the unequal distributions of foreperiods in combination with the zeroing of the data after the end of the foreperiod would lead to differences in the model fits between conditions. However, if this were the case, it should also surface in this control analysis, which it does not.

## Discussion

Here, we have shown that the human EEG tracks temporal hazard, even in a sensory task to which time is not a task-relevant dimension. Forward-encoding models trained to predict the recorded EEG signal from temporal hazard were able to distinguish between experimental conditions that differed by their unfolding of temporal hazard over time. The SMA, a key region in timing and time perception, appeared as the primary source of this tracking signal in a brain-wide search for likely generators of these encoding-model response functions. Our findings show that the mathematical concept of temporal hazard is of use to explain human behavior in an implicit foreperiod paradigm, and that temporal hazard bears tractable signatures in the human EEG. From a methodological point of view, applying the encoding model approach to track a stimulus as abstract as temporal hazard is a novel but promising approach to study temporal processing, as it allows one to map time-resolved processes to the EEG signal.

### Temporal hazard shapes response times

First evidence for the relevance of the concept of temporal hazard to cognitive processing comes from the analysis of response times of the pitch discrimination task. Response times correlated with temporal hazard: the higher the probability of a stimulus to occur at the time point of its occurrence, the faster the response (Fig. 1*E*,*F*; Table 1). This finding replicates the well-established variable-foreperiod effect (Niemi and Näätänen, 1981), which holds that longer foreperiods, genuinely associated to larger hazard, evoke shorter response times.

Furthermore, response time analyses per condition provided empirical evidence that the monotonic and modulated hazard functions affected response time differentially. The relative contributions of the modulated and monotonic hazard functions to the variation of response times changed over conditions (Fig. 1*F*): for the monotonic hazard function, we obtained negative (and significant) weights for all conditions, showing that response times in all conditions decreased with higher values of the monotonic hazard function (i.e., with elapsed time). For the modulated hazard function, we observed a distinction between conditions: we obtained nominally (but not significantly) positive weights for the nonpredictive condition, that is, an increase in response times at the peak of the modulated hazard function, which suggests that the modulated hazard function does not explain well the response times in this condition. For the two predictive conditions, we obtained negative weights that were smaller than those for the monotonic hazard function, showing that the response times decreased at the peak of the modulated hazard function, but also toward the end of the interval where the monotonic hazard function rises. Despite the dominance of the monotonic hazard function in all conditions, these results suggest that the modulated hazard function affected response times differently in the two predictive conditions compared to the nonpredictive condition. Note that, in contrast to the EEG analyses, the response time analyses used one single value from the hazard function for each trial, namely the one at target onset, not the full time-resolved hazard function as used in the EEG analysis, which might not be enough information to distinguish fully between the different levels of temporal predictability.

In sum, response time in all instances is affected by rising temporal hazard due to the passage of time, and additionally captures modulated temporal hazard. This set of results lends validity to our manipulation of temporal hazard (for previous findings in this respect, see Karlin, 1966; Janssen and Shadlen, 2005; Bueti et al., 2010; Todorovic et al., 2015; Tomassini et al., 2016). While previous studies had mostly varied the average foreperiod interval, the present data assert that response time also distinguishes between different degrees of temporal predictability varied around the same average foreperiod.

### Encoding models distinguish between the different conditions of temporal hazard

To test how the brain tracks temporal hazard, we applied an encoding model approach (Lalor et al., 2006, 2009; Naselaris et al., 2011; Mesgarani et al., 2014; Holdgraf et al., 2017) to track temporal hazard in human time-domain EEG, recorded during an auditory foreperiod paradigm (Herbst and Obleser, 2017). The encoding models could distinguish between the different conditions of temporal hazard (see Fig. 5), showing that participants used the implicit variations of foreperiods in the task.

Our results are broadly in line with a number of studies showing that temporal hazard shapes behavior and neural responses in monkeys and humans, underlining the relevance of this mathematical concept in understanding cognitive processes. In monkeys, electrophysiological activity recorded from the lateral intraparietal cortex (area LIP) correlated with temporal hazard during the time of anticipation of the go-signal in a set-go (Janssen and Shadlen, 2005) and a temporal reproduction task (Jazayeri and Shadlen, 2015). Notably, Janssen and Shadlen found that the best-fitting representation of temporal hazard and neural recordings was provided by a smoothed version of the hazard function that takes into account the scalar variability of timing (“subjective hazard”). Applying such a subjective hazard function did not improve model fit in the present data. Probably, this is due to the relative similarity of the hazard functions we used (same mean), as well as the lower signal-to-noise ratio of human EEG compared to single-cell recordings in monkeys.

Further evidence for the relevance of temporal hazard to cognitive processing comes from studies recording fMRI from human participants, providing evidence for a representation of temporal hazard in visual sensory areas during the foreperiod interval (Bueti et al., 2010), and in the responses to the target events in the SMA (Cui et al., 2009). Because of the temporal resolution of the bold signal, these studies used much longer foreperiods. With respect to human EEG, Trillenberg et al. (2000) and Cravo et al. (2011) showed that before target onset, EEG activity distinguishes between different conditions of temporal hazard. Importantly, these designs used strongly discretized foreperiod distributions, with very few foreperiods over all conditions, allowing the comparison of averaged activity over conditions, whereas our approach allowed us to model single-trial data. All of the above-cited studies used a set-go task in which participants had to make a speeded response as soon as the go-target appeared, or even an explicit timing task (Jazayeri and Shadlen, 2015), both of which likely further promoted the use of timing strategies. In contrast, we used a sensory discrimination task, in which the temporal aspects were strictly implicit (for discussion, see also Herbst and Obleser, 2017). Thus, our results show that participants extract the temporal probabilities in the different conditions and use temporal hazard when performing the task.

### Neural signatures of tracking temporal hazard

Importantly, and in complement to the previously published analyses of this data set, the encoding model approach allowed us to directly assess how the human brain encodes temporal hazard for each of the three conditions differing by their hazard function. From the encoding models, we obtained one TRF per condition, whose deflections from zero represent a direct relationship between the EEG time-domain data and the hazard functions, and can thus be interpreted as the neural correlates of temporal hazard.

The monotonic hazard function, describing rising hazard due to the passage of time, revealed the most clear-cut neural signature: a near-instantaneous (i.e., zero-lag) deflection in the response function, peaking at fronto-central sensors. This negative deflection was found in the TRF trained on all three conditions, showing that hazard due to the passage of time is tracked in all conditions regardless of the manipulation of temporal predictability. The sign and sources of this peak, localized to the bilateral SMA and adjacent regions, strongly argues for a relation to the well-described contingent negative variation (CNV; Walter et al., 1964) thought to emerge from the SMA. The CNV has often been described in explicit timing tasks (Macar et al., 1999; Macar and Vidal, 2004; Praamstra and Pope, 2007; Wiener et al., 2010; Merchant et al., 2013; Herbst et al., 2014). Note, however, that we did not observe a clear CNV in the event-related potential analysis presented in Herbst and Obleser (2017), which shows that the encoding model approach exhibits higher sensitivity.

SMA activity, or a CNV, has also been described for implicit timing tasks (Matsuzaka and Tanji, 1996; Akkal et al., 2004; Cui et al., 2009). In monkey electrophysiology, ramping activity in the pre-SMA and SMA during the foreperiod was found when the onset of a target was predictable in time (Matsuzaka and Tanji, 1996; Akkal et al., 2004). Mento et al. (2013) observed a “passive CNV” in an implicit timing task, but reported premotor cortex as its primary source, rather than the SMA, which might be explained by the rhythmic oddball design used in that study (see also Mento, 2013). It is also important to note that neither their study nor ours used individual MRI scans for the source localization, and thus such fine-grained regional divergence should be interpreted with caution. Nevertheless, our findings converge with the literature on temporal processing and provide new evidence that temporal hazard is tracked by the SMA in a strictly implicit timing task.

The sensor-space TRF for the modulated hazard function showed no clear peaks, hence did not allow us to extract a distinct neural correlate of strong predictability. We found a significant difference between the TRF from the nonpredictive and predictive conditions at later lags, which, however, also occurred in the TRF trained with resting state data and therefore seems to be related to the shape of the hazard function rather than the cognitive and neural processing thereof. The source space analysis of the TRF revealed activity in the precuneus around lag zero, only for the strongly predictive condition. This points toward an involvement of the precuneus in temporal prediction, in line with a previous study reporting enhanced precuneus (and default mode network) activation in a rhythmic temporal prediction task (Carvalho et al., 2016). Extracting only the data from the SMA in a *post hoc* analysis revealed a positive-to-negative deflection around lag zero for the TRF of the modulated hazard function in the strongly predictive condition only (see Fig. 6*B*,*C*), which tentatively suggests that the SMA also processes the modulation of temporal hazard in that condition in an instantaneous way, but might not be the dominant region to do so.

In sum, the TRF for the monotonic hazard functions revealed a quasi-instantaneous neural correlate of tracking hazard, localized in the SMA, whereas the TRF for the modulated hazard functions revealed no singular feature well defined in space and time. In sum, our findings converge with the literature on temporal processing and provide new evidence that temporal hazard is tracked by the SMA in a strictly implicit timing task. The successful distinction between conditions by the encoding models might thus result more from the decreasing applicability of the monotonic hazard function over conditions (in line with the by-trend stronger negative peak for the nonpredictive condition) than from the distinct encoding of the predictive hazard function.

### Methodological relevance of the encoding model approach to abstract, time-resolved stimuli

Our results reveal the usefulness of the forward-encoding model approach applied to a seemingly abstract, mental representation such as temporal hazard. The approach has previously been applied successfully to encode the attentional processing of sensory stimuli (Ding and Simon, 2012; O’Sullivan et al., 2015; Fiedler et al., 2017; Puvvada and Simon, 2017) and semantic processing of speech streams (Broderick et al., 2018; Di Liberto et al., 2018), but never to a purely mental stimulus such as temporal hazard. Modeling EEG data with a time-resolved regressor allows us to study how the brain maintains representations of complex and dynamic features such as temporal hazard. Furthermore, the approach of computing TRF for single trials can take into account only the foreperiod interval during which timing is expected to occur, between the cue and the target onset. These intervals vary over trials and conditions, which is a challenge for standard approaches of EEG data analyses involving averaging.

To validate our approach, we have provided two control-level encoding models. One is for the sensory stimulus from the EEG data recorded during the task to show that the encoding model approach is sensitive: TRF (Fig. 3) to auditory target onsets replicated target-evoked event–related potentials shown previously for these data (Herbst and Obleser, 2017). The second control-level encoding models used the temporal hazard of interest as regressor but were trained on resting-state instead of the actual task data (Fig. 7). Thus, the encoding model approach is also specific, in that it fails when applied to surrogate or unrelated data. The shapes of the TRF computed on these resting-state data show that the regressors used for the encoding model partially affect the TRF, calling for caution in the interpretation of their shape without performing a model comparison procedure.

## Conclusions

Using an implicit probabilistic foreperiod paradigm, forward-encoding models have allowed us to quantify the tracking of temporal hazard in the human EEG. Our results suggest that the neural correlates of modulated temporal hazard only partially overlap with those of monotonically rising hazard due to the passage of time. The monotonic hazard function revealed a quasi-instantaneous neural correlate of tracking hazard, whereas the modulated hazard function revealed no singular feature well-defined in space and time. Source localization and a set of control analyses capture the functional and anatomic specificity of these effects, with a key role for the SMA. The model-driven approach used here illustrates how implicit variations in temporal hazard bear tractable signatures in the human electroencephalogram.

10.1523/ENEURO.0017-18.2018.ed1 Extended Data 1 The Matlab and R analysis code used for the analyses of the EEG and behavioral data are available at he Open Science Framework (https://osf.io/qbhma/). The zip-archive *ExtendedData1* contains the R scripts for the analysis of response times, Matlab scripts for the encoding models computed on the task- and resting-state data, and the Matlab scripts used for the source localization. Download Extended Data 1, ZIP file.

## Synthesis

Reviewing Editor: Tatyana Sharpee, The Salk Institute for Biological Studies Decisions are customarily a result of the Reviewing Editor and the peer reviewers coming together and discussing their recommendations until a consensus is reached. When revisions are invited, a fact-based synthesis statement explaining their decision and outlining what is needed to prepare a revision will be listed below. The following reviewer(s) agreed to reveal their identity: Ana Todorovic, Martin Wiener

The study tests a novel methodological approach for assessing anticipation of stimulus events prior to onset of the stimulus. The review comments provided below in particular identify the need to clarify statistical analyses methods and how particular electrodes were selected for the analysis.

Reviewer 1:

In their manuscript “Tracking temporal hazard in the human electroencephalogram using a forward encoding model”, authors apply a forward encoding model to a temporal expectation paradigm. Participants anticipated the onset of tones in order to respond, where the distribution of tone onset times differed per block. In one type of block, onsets were equiprobably distributed (leading to an exponential increase in expectation as the trial unfolds), while in other blocks the maximal probability of occurrence was in the middle of the trial (leading to an additional peak mid-way through the trial). The authors conclude that it is possible to track this anticipatory, pre-stimulus activity by separately modelling two hazard functions: one that tracks expectation as a function of elapsed time, and one that independently captures (in the case of the bell-shaped tone onset blocks) the shape of the distribution of onset times. They find that only the first hazard function is related in a clear-cut way to both behavioural responses and neural activity, and is localized to the SMA.

The study was carefully conducted and the methodological approach is sound, including a very nice control analysis on resting state data. I have no major concerns with the manuscript.

Coming from a cognitive perspective, I had difficulty untangling the methodological approach from the cognitive conclusions. On the one hand the central problem outlined in the abstract is cognitive (“here, we tackle the question of whether and how the human brain tracks continuous temporal hazard...”), on the other hand the discussion largely focuses on the methodological approach. Personally, I would have appreciated more of a return to cognition / neural activity after the results were outlined.

For example, one of the results was that the temporal response function of the modulated hazard distinguishes between the conditions at a small negative lag, and a somewhat larger (150 ms) positive lag. What might this mean, in terms of anticipating an incoming stimulus? Could the positive lag correspond to heightened neural activity to non-occurrence of expected tones? Why would the predictive value be stronger for the nonpredictive condition here (Figure 4B, bottom)? It would be good to see some speculation about this in the Discussion.

Additionally, some parts of the Discussion keep to language better suited for the Results section. For example, the relation of hazard functions and response times are described in terms of the sign of the regression weights rather than whether people are quicker to respond at certain time points. (End of second paragraph under “Temporal hazard shapes response times”, page 19). Another example, when discussing neural signatures of tracking temporal hazard (page 21), it says that “extracting only the data from the SMA revealed a positive deflection at negative lags” (etc), but this is again not unpacked into what it might mean for cognitive processing, neural activity or behaviour. Overall, it might be a good rule to keep coming back to the question of what each of the results tells us about anticipating events in time.

I was also wondering about the process of predicting trials in a condition based on all the other trials. Would the unequal tone onset distribution in the two bell-shaped blocks (and the bringing the signal to zero after tone onset in each trial) perhaps result in unequally good predictions across the length of the epoch? (i.e. worse predictions for trials with short and long foreperiods because of skewed representations towards the most common, middle foreperiod trials.) Is this relevant for the later condition comparisons?

Finally (and this is very minor), it was unclear to me whether the catch trials were included in the calculations of the hazard functions. They seem to be included in Fig 1B, so I would guess yes, but it would be good to explicitly mention this.

I sign my reviews,

Ana Todorovic

Reviewer 2:

Herbst and colleagues present a re-analysis of a previously collected EEG study, in which subjects (N=24) were required to perform a pitch discrimination task. The critical manipulation in this task was the interval of time from trial onset to tone onset (foreperiod), which varied from trial to trial in either a uniform manner or a Gaussian one, with the latter having two separate widths; all three foreperiod distributions had the same mean.

Behavioral data revealed that response times for the pitch task varied according to the foreperiod distributions that were presented. For all distributions, response times decreased with longer foreperiods; however, with Gaussian distributions, where the foreperiod on average occurred around the mean, response times indicated an additional benefit at this point. Regressing response times with the hazard function probability of occurrence for each distribution revealed that lower response times also occurred at higher hazard function probabilities. When comparing conditions, uniform hazard functions provided strong predictive strength for response times across all three conditions, whereas the hazard function for Gaussian forepreriods did not significantly predict response times, even though the association did differ between conditions.

For EEG data, in addition to auditory responses linked to temporal cortices, regression of the hazard function for the uniform foreperiod distribution onto EEG data revealed an early effect in the signal that was predictive of the monotonic rise in hazard rate. For the hazard function for Gaussian foreperiods, a sustained effect was observed, but in a control analysis with resting-state data this finding was also observed, suggesting a spurious connection. In two additional analyses, the authors went on to link the uniform distribution hazard rate to activity in the SMA, and also find that the EEG regression models were able to recapitulate the observed EEG data to a satisfactory degree. The authors conclude that indices of the hazard rate are present in EEG data.

The behavioral results of the study are interesting, but I am not entirely convinced that the EEG data provide much else. It appears that the data as (re)analyzed shows, as the behavioral data do, that a hazard rate from a uniform distribution provides a dominant effect over a hazard rate from a Gaussian one, in that only the uniform distribution can provide any meaningful data (see below).

MAJOR:

-In the correlation analysis of the predictiveness of the regression model, the authors note that the non-predictive and strongly-predictive data exhibit correlations in the opposite directions, as would be predicted, and that these were significantly different from one another, but this does not necessarily suggest that the models at all were accurate for what they were predicting. Indeed, the authors here appear to be demonstrating a sort of cross-validation for their technique. Here, the correlation coefficients being reported are very low (r=0.07 on average) and may not even be greater than chance. Beyond a simple comparison with a null point, the authors do have the control comparison with resting-state EEG, but have not compared their correlations between the two. For example, does the non-predictive correlation with EEG task data significantly exceed the correlation with EEG resting data?

-While the source-space analysis is interesting, I'm skeptical of it for several reasons. First, the authors used no method that I can see to constrain the inverse solution on an individual basis, as individually digitized electrode locations were not used and no anatomical scans were available. Second, the authors appear to conduct a circular analysis by localizing the peak of the TRF to the SMA, and then extracting the time series and conducting the same analysis. Third, how were the data extracted for this region? Are the data shown/analyzed from a single voxel, or from the collection of significant voxels in this region, or the entire (atlas-defined) region?

-The EEG signal-space analysis also exhibits some issues. Global field power and frequency-space analyses are both used, but had not been mentioned previously in the methods, and it is unclear why they were examined (unless this was intended as an exploratory analysis to see what signals, if any, might connect to hazard rates).

-The authors would do well to contextualize their findings a bit more in the discussion. Previous work has examined if alpha oscillations match temporal predictions in foreperiod designs (Samaha, et al. 2015; Bablioni, et al. 2004), and other work has demonstrated that the CNV automatically tracks temporal durations, even when subjects are not explicitly instructed to do so (Mento, et al. 2013).

-Most notably, this paper is a re-analysis of previous work (Herbst & Obleser, 2017), and it is unclear what this manuscript adds beyond the earlier publication, which was very thorough in its analysis and dissection of the findings (including the behavioral effects). The only major difference is the linear regression of hazard rate onto EEG (and behavioral) data, along with the source analysis, the results of which are not totally clear. The authors should at least dissociate their findings here from the earlier work.

MINOR:

-How were noisy data assessed? What was the average number of channels/trials rejected per subject?

-The authors use the term ‘training’ for comparing fits with actual data in a leave-one-out manner, but this isn't necessarily accurate. As far as I can tell, the model was not trained so much as fit via ridge regression, and then cross-validated. If the authors had used a machine-learning technique to train a classifier, then this term would be more appropriate.

-The authors describe ‘explicit’ vs ‘implicit’ timing strategies to distinguish their study from others. It would be helpful to explain these terms better and provide appropriate citations (i.e. Coull & Nobre, 2008).

-What was the result of the 10% trials where they were explicitly asked about the foreperiod?

-What was the nature of the catch trials? How much longer/shorter than the mean of each distribution were they?

-In general, I recommend making the figures larger. There are many panels and much of it was difficult to reach and interpret. Given the online format of the journal, more space can be devoted to the various parts.

-Page 5: The authors note that the hazard function regressors had to match the EEG sample rate, and so they interpolated missing values in the regressor. Was this necessary? Why not use the same number of time points for t in equation 1?

-For each of the distributions, was the foreperiod duration drawn randomly on each trial? Or were the durations presented counterbalanced such that subjects experienced each possible duration at least once?

-Figure 1F: Please label the x-axis. Does each column refer to one of the presented foreperiod durations?

-Figure 2: please replace ‘convoluting’ with ‘convolving’

-Figure 4: different electrodes are displayed in panel C for the TRF (Fz at top and Cz at bottom) why is this? Were statistics run on separate electrodes? In the bottom right, three topoplots are displayed for the 7-8Hz effect; are these for the three different lags? Or the three different distributions?
